# CEO turnover and corporate innovation: What can we learn from Chinese listed companies

**DOI:** 10.3389/fpsyg.2022.874907

**Published:** 2022-07-20

**Authors:** Shujun Sun, Haiwei Jiang

**Affiliations:** ^1^School of Economics, Zhejiang University, Hangzhou, China; ^2^School of International Trade and Economics, Central University of Finance and Economics, Beijing, China

**Keywords:** CEO turnover, corporate innovation, quality of innovation, top management team reorganization, research and development

## Abstract

Using data of China’s listed companies from 2000 to 2016, we employ a staggered difference-in-difference (DID) approach to identify the causal effects of CEO turnover on corporate innovation. First, we find that listed companies with CEO turnover experienced an average increase of 9.5% in the quantity of innovation and 8.9% in innovation quality after the change. The dynamic effect test supports the parallel trend condition, and the placebo test rules out the nonrandom selection issue. Second, the heterogeneity tests show that CEO turnover plays a more prominent role in promoting innovation for listed firms with CEO duality, high financial constraints, and in high-tech industries. Third, CEO turnover affects corporate innovation by driving top management team reorganization and promoting R&D input. This paper has important implications for the understanding of the role of CEO turnover in companies’ innovation, as well as for strategy formulation and implementation.

## Introduction

Deepening the reform of corporate governance and promoting innovation ability is a key to promoting a company’s business performance. Entrepreneurship is a critical driving force in promoting corporate innovation (e.g., Schumpeter, 1942; Decker et al., 2014). Entrepreneurs have unique characteristics in cognitive ability, discovery ability, ability to take advantage of market opportunities, and coordination of professional knowledge. This uniqueness urges entrepreneurs to constantly try the combination of new structures and new requirements to optimize resource allocation to obtain technical and market advantages ahead of their competitors. Moreover, entrepreneurship is a process by which the economy moves forward through the act of creative disruption or innovation. Thus, entrepreneurs are key agents of innovation and creative destroyers (Schumpeter, 1934).

As an essential part of corporate governance, the CEO is a decisive policy-maker and an executive of a company, playing a crucial role in the enterprise development strategy (Beatty and Zajac, 1987). Some enterprises can gain strong market power and global competitiveness because CEOs attach great importance to innovation investment (Islam and Zein, 2020). For example, Watts was responsible for Apple Inc. in the early stage. Later, the new CEO, Steve Jobs, developed a number of novel products, such as Apple II, making this company well-known and profitable. Jobs was a CEO who paid great attention to innovation, and believed that the difference between leaders and followers in the market lies in innovation.

We attach attention to the role of CEO turnover in corporate innovation in China since there are an increasing number of innovative firms and start-ups whose CEO plays a determinant role in innovation strategy. For example, the number of patent applications of Huawei ranks first among global enterprises in 2019. Huawei’s 45% employees are engaged in research and development (R&D), and it has insisted on investing at least 10% of its annual sales in R&D since 1992. Zhengfei Ren, its CEO, believes that although innovation is difficult and complex, it is the only way for enterprises to survive and succeed. Another point is that the CEO is obliged to take responsibility for innovation strategy. Therefore, Huawei has created the “rotating CEO” system in terms of enterprise decision-making mechanisms. Specifically, seven vice presidents take turns to serve as the CEO for every 6 months, which enables inventors to try and practice new ideas constantly, and trains many outstanding talents and inventors for the company.

This paper focuses on technological innovation instead of management innovation which is also a fundamental part of innovation (Rajiani and Ismail, 2019), and studies the relationship between CEO turnover and innovation performance in China by employing a staggered DID design. Specifically, we compare innovation output between companies with CEO turnover and companies without any CEO turnover before and after the change. We find that compared with the listed companies without CEO turnover in China, the quantity and the quality of innovation of the listed companies with CEO turnover increase by 9.5 and 8.9%, respectively. Furthermore, we show that CEO turnover promotes the reorganization of the senior management team, and enhances R&D investment as well.

The rest of this paper is organized as follows. Section 2 reviews the related literature and develops research hypotheses. Section 3 introduces data and variables for this study. Section 4 reports the identification strategy and the empirical results. Section 5 conducts additional tests to confirm the robustness of the main results, and discusses the heterogeneity effects. Section 6 examines the potential mechanisms. Section 7 concludes.

## Literature and hypotheses

A bunch of studies believe that CEO turnover can promote enterprise innovation. Aghion et al. (2013) and Sapra et al. (2014) find that incumbent managers are unwilling to bear the risks and changes brought about by innovation. Therefore, CEOs will reduce R&D investment in the period before they leave the enterprise to avoid risks and obtain short-term benefits steadily (e.g., Murphy and Zimmerman, 1993). However, due to stricter supervision of investors and the crisis of potential acquisition, the new CEOs tend to divest poor-performing projects, and allocate resources to the more promising investment opportunities, thus likely leading to more effective innovation (Weisbach, 1995; Huson et al., 2004). Additionally, the enterprise will give the new CEO more flexibility to adjust the enterprise’s major decisions. In that case, the new CEO is more likely to take various measures to increase R&D investment, optimize innovation strategies, and improve the company’s innovation ability (Bereskin and Hsu, 2014). Faleye et al. (2014) show that after CEO turnover, the firms with better-connected CEOs invest more in R&D and receive more and higher quality patents. An interquartile change in CEO connections is associated with increases of 9.7% in R&D investment, 11% in patents, and 4% in citations. This leads to our first hypothesis,

*H1a*: CEO turnover improves a firm’s innovation.

Some research believes that CEO turnover will negatively impact corporate innovation. It is found that CEO turnover may disrupt the original long-term plan of the enterprise, which can impede the innovation process (Clayton et al., 2005). The successor CEO needs a long period to understand the company’s previous business model, and adopts a relatively stable strategy instead of developing new innovation projects blindly since successors who are under-prepared and reluctant to adopt risky projects with poor performance will be fired by the board (Datta and Guthrie, 1994; Denis and Denis, 1995). According to such studies, we propose the following hypothesis,

*H1b*: CEO turnover impedes a firm’s innovation.

Overall, from the empirical literature, there are debates on how CEO turnover affects corporate innovation. In particular, there was very little literature to explore the relationship between CEO turnover and corporate innovation in China. This paper contributes to the existing research in two aspects. First, we manually sort out the CEO turnover event of Chinese listed companies, and identify the causal effect of CEO turnover on corporate innovation in China (e.g., Clayton et al., 2005; Faleye et al., 2014). Second, taking the enterprise innovation output as a measure, this paper systematically investigates the impact of CEO turnover on both the quantity and quality of corporate innovation through data matching. It obtains some new research results, which makes up for the deficiency that the prior literature only pays attention to the quantity of corporate innovation.

The existing literature points out that technological innovation must be integrated with management innovation within organizations, such as adopting new organizational structures (Rajiani and Ismail, 2019). According to the theory, CEO turnovers can be categorized into three types: internal follower succession, internal competitor succession, and external succession. Internal follow-up successors are expected to avoid excessive restructuring since they have similar behavioral inertia with the former CEO. For internally competing successors, the conflict of strategic concepts and the frustration of power struggle often lead to team reorganization shock. For external successors, to consolidate their power, some former executives will be promoted or excluded, which is more likely to aggravate the tension among the senior management team members and lead to team shock. It is believed that the re-construction of senior management team is the crucial variable of the company’s strategic change, which will impact the sequential innovation. Specifically, after the CEO turnovers, the new CEO is more likely to promote the reorganization of the senior management team. The reorganized senior management team is full of fresh blood, bringing new ideas, strategies, and vitality (Wiersema and Bantel, 1992) and positively affecting innovation (Bantel and Jackson, 1989). Furthermore, Riana et al. (2020) find that human resource management is a determinant of firm innovation and performance. Based on the above discussion, we then develop the following hypothesis:

*H2*: CEO turnover affects a firm’s innovation through reorganization of the senior management team.

After the CEO change, the board strengthens the supervision of the successor CEO, to ensure the efficient operation of the enterprise (Karaevli and Zajac, 2013). Since innovation is a determinant of enterprise profits, it is generally a vital work of the new CEO to boost research activities. Some studies find that the new CEO will increase R&D investment after succession to improve the innovation capacity (e.g., Bereskin and Hsu, 2014). As innovation culture and collaboration play a crucial role in conducting innovation (Ab Rahman et al., 2018; Arsawan et al., 2020), adjustment of R&D input can promote innovation by shaping proper innovation strategy. This paper believes that this channel also exists in China. That is, after the CEO turnover, the new CEO pays more attention to R&D input, to promote innovation ability and thus improve production efficiency and operating profits. Therefore, we establish the following hypothesis:

*H3*: CEO turnover affects a firm’s innovation by adjusting the strategy of R&D investment.

In the understanding of educational psychology and innovation, this paper makes two contributions to the extant studies. First, we complement the literature concerning the relationship between management and innovation (e.g., Riana et al., 2020) by documenting that reorganization of senior management team is an important channel between CEO turnover and innovation. Second, we find that adjustment of R&D input after CEO turnover is beneficial to innovation, which provides suggestive evidence to back up a strand of studies investigating the linkages of innovation culture and innovation strategy to innovation (e.g., Ab Rahman et al., 2018; Arsawan et al., 2020).

## Data

### Data sources

The research object of this paper is all listed companies of the main board in China from 2000 to 2016. CEO personal information and financial data of listed companies are compiled from the CSMAR, WIND, and CNINFO databases, which provide comprehensive data for China’s listed firms. Following He et al. (2018), this paper matches the Chinese patent database from the State Intellectual Property Office (SIPO) with the firm-level data to obtain the patent information of listed companies. Following previous studies (e.g., Fang et al., 2017), we drop the observations whose key financial indicators are missing. All variables are winsorized at the 1st and 99th percentiles to eliminate the effect of outliers. Finally, we obtain 15,151 observations of 2,665 listed companies.

### Variables

#### Measuring innovation

Following extant studies (e.g., He and Tian, 2013; Chen et al., 2022; Jiang et al., 2022), we measure the innovation performance of listed companies from two aspects: innovation quantity and innovation quality. Patents can be divided into substantive innovation and strategic innovation according to the difference of innovation motivation. Invention patents are substantive innovation that can promote technological progress, while utility model and design patents are strategic innovation which is produced to meet the policy. Therefore, invention patents can truly measure the level of corporate innovation. This paper takes the number of invention patent applications, *Invention*, to measure the innovation quantity of listed companies. And we take the number of forward patent citations received by companies, *Citation*, as the measure of the innovation quality. As the distribution of patent applications and patent citations is right-skewed, we use the natural logarithm of two variables. To avoid losing observations with zero patents or citations, we add one to the actual values when calculating the natural logarithm.

#### CEO turnover

As a general practice in the literature (e.g., Clayton et al., 2005), we compare the names of CEO of each company in order. If names in two consecutive years differ, it is identified that CEO turnover has occurred and otherwise has not. The explanatory variable, *Turnover*, is a binary variable that equals one for years in and after the year of initial CEO turnover, and zero otherwise. The timing of the CEO turnover event of listed companies in our sample is different so that we can design a staggered DID model for the empirical analysis.

#### Control variables

Following the literature on corporate finance and corporate innovation (e.g., Chang et al., 2015), the control variables, which may affect innovation, collected in this paper are composed of two parts. The first part is the control variables related to the enterprise’s financial indicators, including total asset net profit margin (*ROA*), enterprise age (*Age*), asset-liability ratio (*Leverage*), enterprise size (*Size*), enterprise ownership (*SOE*), enterprise growth (*TobinQ*), board of directors (*Board*), the proportion of independent directors (*Director*) and board of supervisors (*Supervisor*); The other part is the control variables related to the CEO’s characteristics, including whether the CEO concurrently serves as the chairman (*Duality*), CEO’s age (*CEO_age*) and CEO’s education background (*CEO_edu*).

#### Descriptive statistics

Table 1 reports the descriptive statistics of main variables, including variable name, mean, standard deviation, and variable definition. It can be seen that there are many CEO turnovers of China’s listed companies during the sample interval, with a mean of 0.882. The mean invention patent applications of listed companies are 1.371, and the mean patent citations received of innovation are 0.810. The mean age of the enterprises in the sample is around 13 years, and the state-owned enterprises account for about 45%. The size of the board of directors is about 9, and the proportion of independent directors is about 37%. CEOs are 48 years old on average, and 84% of them have a bachelor’s degree or above. These results align with the actual situation, which shows that this paper’s selection of control variables is reasonable. The pairwise correlations of major variables at the firm level in our analysis are reported in Appendix Table A1.

**Table 1:** Variable definition and descriptive statistics.

Variables	Observations	Mean	S.D.	Definition
Invention	15,151	1.371	1.448	The number of invention patent applications plus 1, then take the logarithm
Citation	15,151	0.810	0.818	The number of invention patents cited plus 1, then take the logarithm
Turnover	15,151	0.882	0.323	If the CEO changes, take 1, otherwise take 0
ROA	15,151	0.044	0.049	Net profit / total asset balance
Age	15,151	13.169	5.321	Year of observation minus year of establishment
Leverage	15,151	0.424	0.209	Total liabilities / total assets
Size	15,151	21.843	1.215	Logarithm of total assets
SOE	15,151	0.448	0.497	Take 1 for state-owned enterprises and 0 for non-state-owned enterprises
TobinQ	15,151	2.842	2.011	Market value / total assets
Board	15,151	8.988	1.839	Number of directors
Indirector	15,151	0.365	0.058	Number of independent directors / directors of board
Supervisor	15,151	3.773	1.199	Number of supervisors
Duality	15,151	0.237	0.425	1 for CEO concurrently serving as chairman, otherwise 0
CEO_age	15,151	48.258	6.302	CEO’s age
CEO_edu	15,151	0.844	0.363	1 for bachelor degree or above and 0 for others

## Empirical analysis

This section describes our empirical strategy, and reports the regression results. Then, we carry out the common trend test, and placebo test to examine the precondition of DID model.

### Empirical strategy

Due to staggered CEO turnovers for different companies, this paper employs the DID model to investigate the impact of CEO turnover on innovation performance. The benchmark econometric model is as follow:


(1) $$\begin{array}{c} \mathrm{Innovatio}n_{\mathrm{ft}}=\alpha +\beta \cdot \mathrm{Turnove}r_{\mathrm{ft}}+\delta^{\prime }\mathrm{FirmControl}s_{\mathrm{ft}}+\\ \gamma^{\prime }\mathrm{IndividualControl}s_{ift}+\eta_{f}+\eta_{t}+\eta_{i}+\varepsilon_{\mathrm{ft}} \end{array}$$

where *f* denotes the listed company, *i* denotes the CEO and *t* denotes the year. The dependent variable $\mathrm{Innovatio}n_{\mathrm{ft}}$ represents the innovation performance of listed companies, including innovation quantity and innovation quality. The core explanatory variable $\mathrm{Turnove}r_{\mathrm{ft}}$ indicates whether the CEO of a listed company has been changed, and its corresponding coefficient *β* is the key interest of this paper, capturing the impact of CEO turnover on innovation. $\mathrm{FirmControl}s_{\mathrm{ft}}$ is a vector of control variables at the firm level to control the factors potentially associated with innovation performance. $\mathrm{IndividualControl}s_{ift}$ is a vector of control variables at the CEO’s level to balance CEOs’ characteristics before and after CEO turnover. *η*_f_ is the fixed effect of enterprises to control the time-invariant characteristics of enterprises. *η_t_* is the time fixed effect to control the time-varying shocks. *η_i_* is the CEO individual fixed effect, which controls the time-invariant CEO characteristics. $\varepsilon_{\mathrm{ft}}$ is the stochastic error term. Due to the possible autocorrelation of innovation performance over time, all econometric models use the standard error to have arbitrary heteroskedasticity and autocorrelation by clustering standard errors at the firm level (Blanco and Wehrheim, 2017).

### Baseline regression

This paper uses the stepwise regression method to estimate the benchmark model to study the impact of CEO turnover on the quantity and quality of innovation, and the estimation results are shown in Table 2. Columns (1)–(3) report the regression results with respect to innovation quantity. The R-squared value of each column is greater than 0.7, which suggests that the data fit the regression model well. Column (1) controls enterprise fixed effect and time fixed effect, showing that the estimated coefficient is positive and statistically significant at the 1% level. Column (2) adds the firm-level control variables, and the estimated coefficient is positive and statistically significant at the 1% level. Column (3) additionally adds CEO personal control variables and individual fixed effect. The estimated coefficient is 0.095, which means CEO turnover positively affects the innovation quantity of listed companies at the 1% level. Columns (4)–(6) report the regression results with respect to innovation quality. The R-squared value of each column is greater than 0.5, indicating that the model explains more than 50% of the fitted data in the regressions. Column (4) controls the listed companies’ fixed effect and time fixed effect, column (5) further controls the firm-level control variables, column (6) additionally controls the CEO’s control variables and CEO’s individual fixed effect as well. The estimations show that the impact of CEO turnover on innovation quality is positive and statistically significant at the 1% level. Overall, the baseline regression results provide consistent evidence to support Hypothesis 1a that CEO turnover strengthens the quantity and quality of firms’ innovation.

**Table 2:** Innovation quantity and innovation quality.

	(1)	(2)	(3)	(4)	(5)	(6)
Dependent variables	Invention	Invention	Invention	Citation	Citation	Citation
Turnover	0.130^***^	0.103^***^	0.095^***^	0.077^***^	0.071^***^	0.089^***^
	(0.034)	(0.034)	(0.034)	(0.023)	(0.023)	(0.028)
Size		0.429^***^	0.520^***^		0.082^***^	0.095^***^
		(0.043)	(0.050)		(0.021)	(0.027)
SOE		0.186^**^	0.192^*^		0.046	0.064
		(0.086)	(0.105)		(0.051)	(0.067)
ROA		−0.652^**^	−0.237		−0.184	−0.130
		(0.272)	(0.302)		(0.182)	(0.209)
Age		0.042	0.037		0.002	0.012
		(0.040)	(0.038)		(0.022)	(0.031)
Leverage		−0.033	−0.074		−0.010	0.025
		(0.119)	(0.133)		(0.064)	(0.079)
TobinQ		0.016^**^	0.012		0.001	0.003
		(0.008)	(0.009)		(0.005)	(0.006)
Board		−0.176	0.004		−0.354^**^	−0.008
		(0.284)	(0.014)		(0.166)	(0.009)
Indirector		0.001	−0.146		−0.003	−0.300
		(0.024)	(0.319)		(0.015)	(0.201)
Supervisor		0.001	0.017		−0.003	0.014
		(0.024)	(0.027)		(0.015)	(0.018)
Duality			−0.039			−0.025
			(0.055)			(0.040)
CEO_age			0.000			−0.020
			(0.027)			(0.019)
CEO_edu			−0.018			−0.029
			(0.095)			(0.063)
Constant	1.256^***^	−8.836^***^	−10.676^***^	0.743^***^	−0.949^*^	−0.438
	(0.030)	(1.069)	(1.825)	(0.020)	(0.544)	(1.159)
Firm fixed effect	Yes	Yes	Yes	Yes	Yes	Yes
Time fixed effect	Yes	Yes	Yes	Yes	Yes	Yes
Individual fixed effect	No	No	Yes	No	No	Yes
R-squared	0.722	0.733	0.724	0.530	0.531	0.458
Observations	15,151	15,151	15,151	15,151	15,151	15,151

Robust standard errors in parentheses are clustered by the listed company. ^***^, ^**^ and ^*^ denote significance at the 1, 5, and 10% level, respectively.

This paper prefers to select columns (3) and (6) as the baseline regression results. The estimation results show that CEO turnover has a statistically significant and positive average treatment effect (ATE) on the innovation of listed companies. In particular, compared with the sample without CEO turnover, the innovation quantity of listed companies with CEO turnover increases by 9.5%, and the quality of innovation increases by 8.9% on average, after the event. For control variables, there is a positive correlation between enterprise scale and innovation quantity and innovation quality at the 1% level, and there is a positive correlation between ownership of state-owned enterprises and innovation quantity at the 10% level, which is consistent with the literature (e.g., Hu et al., 2017).

### Dynamic effect test

The validity for the above DID model relies on the parallel trend assumption that the treatment group (with CEO turnover) and the control group (without CEO turnover) do not have a statistically significant difference in innovation performance before the change. In order to examine this precondition, following Beck et al. (2010), we use the event study method to study the dynamic effect of CEO turnover on corporate innovation. We consider a 15-year window, from 5 years before the CEO turnover to 10 years after the CEO turnover, and set the following regression equation:


(2) $$\begin{array}{c} \mathrm{Innovatio}n_{\mathrm{ft}}=\alpha +\beta_{1}\mathrm{Turnove}r_{\mathrm{ft}}^{-5}+\beta_{2}\mathrm{Turnove}r_{\mathrm{ft}}^{-4}+\ldots +\\ \beta_{15}\mathrm{Turnove}r_{\mathrm{ft}}^{+10}+\gamma^{\prime }\mathrm{IndividualControl}s_{ift}+\\ \delta^{\prime }\mathrm{FirmControl}s_{\mathrm{ft}}+\eta_{\mathrm{ft}}+\eta_{t}+\eta_{i}+\varepsilon_{\mathrm{ft}} \end{array}$$

where $\mathrm{Turnove}r_{\mathrm{ft}}^{-j}$ means the *j* years before the initial CEO turnover, and $\mathrm{Turnove}r_{\mathrm{ft}}^{+j}$ means the *j* years after the initial CEO change, both of which are dummy variables. The model is estimated with the 1 year before the CEO turnover as the benchmark year.

**Figure 1:**
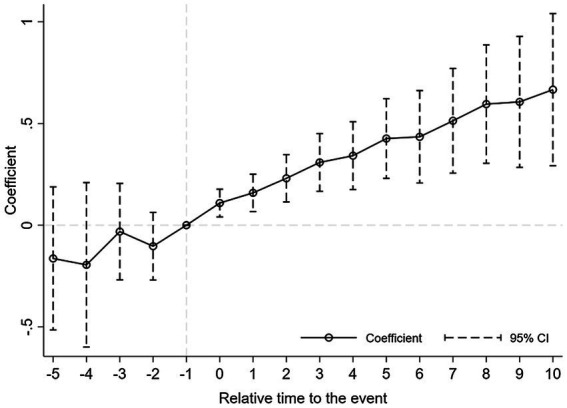
Dynamic effect - invention.

**Figure 2:**
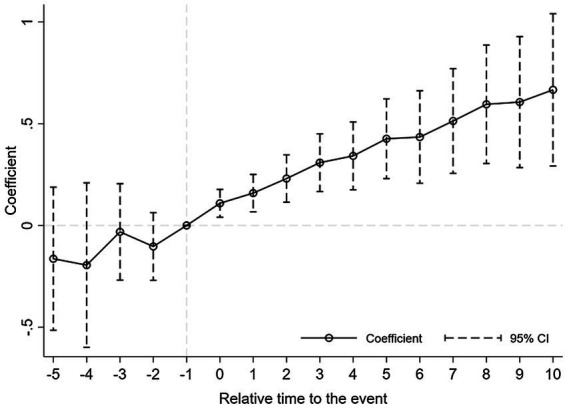
Dynamic effect - citation.

The dynamic effect test results (i.e., dummy variable estimation coefficients) are reported in Figures 1, 2. The result shows no statistically significant difference in the quantity and quality of innovation between the treatment group and the control group before the change event, which meets the parallel trend condition. In addition, CEO turnover affects the quantity and quality of innovation immediately, and has a long-term positive impact. Besides, the effects of CEO turnover on the innovation quantity and quality continue to increase over time.

### Placebo test

**Figure 3:**
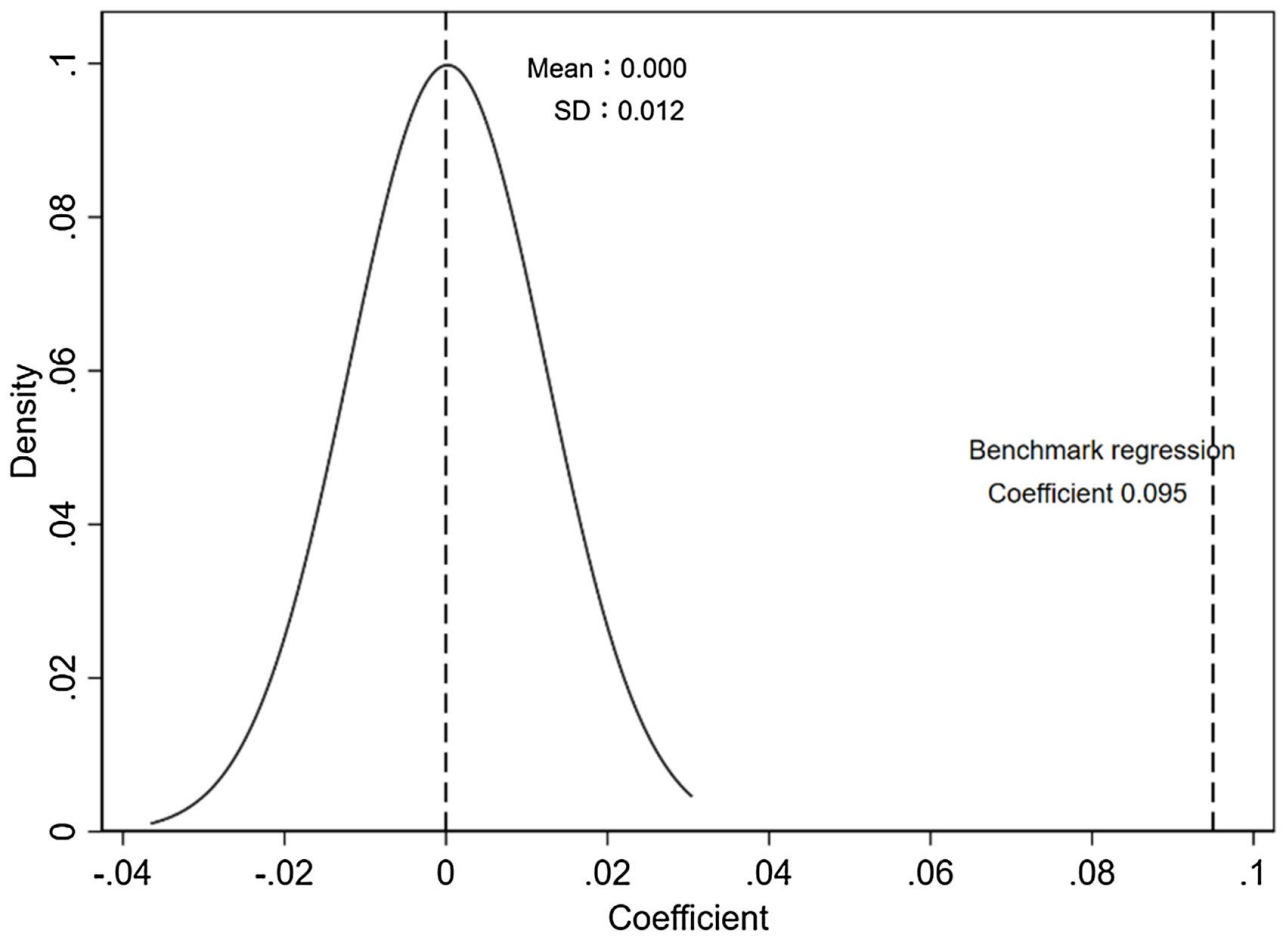
Placebo test - invention.

**Figure 4:**
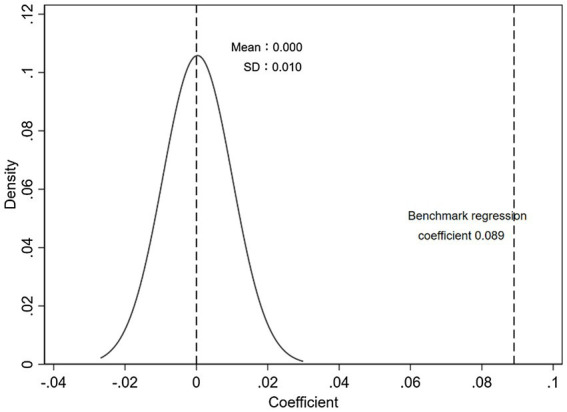
Placebo test - citation.

We implement placebo tests to rule out the possibility that the baseline results are purely driven by chance. We draw a random sample to re-estimate the baseline model according to the number of observations of the original treatment group and control group. We repeat this procedure 500 times, and the distributions of the estimated coefficient are reported in Figures 3, 4. The results show that the regression coefficient obtained by randomly sampling is close to 0, while the actual regression coefficients from Table 2 are outside the 99.73% (3 standard deviations) confidence interval, which excludes the possibility of baseline results caused by chance.

## Further discussion

First, we implement the PSM-DID method to deal with the problem of sample selection. In addition, this paper further controls some other confounding factors of enterprise innovation to test the robustness of baseline results in Appendix Tables A2 and A3, including firm-, industry-, and region-level characteristics. Second, we discuss the heterogeneities of the impact of CEO turnover on corporate innovation.

### PSM-DID

In the parallel trend test, we find no significant difference in innovation performance between the treatment group and the control group before the CEO turnover event. In order to further reduce the sample selection biases caused by the different initial conditions of the treated group and the control group, we employ the PSM-DID method to examine whether our baseline DID results are robust. Following He and Tian (2013), we select firm-level control variables in the benchmark regression as covariates, and the samples of treatment group and control group are matched one to three. The balance test of covariates is shown in Appendix Figure A1, and the descriptive statistics of explanatory variables is shown in Appendix Table A4. The matched samples are used for estimation, and the regression results are reported in Table 3.

**Table 3:** PSM-DID.

	(1)	(2)
Dependent variables	Invention	Citation
Turnover	0.124^**^	0.124^***^
	(0.056)	(0.045)
Firm control variables	Yes	Yes
Individual control variables	Yes	Yes
Firm fixed effect	Yes	Yes
Time fixed effect	Yes	Yes
Individual fixed effect	Yes	Yes
R-squared	0.871	0.759
Observations	6,457	6,457

A constant term is included but not reported. Robust standard errors in parentheses are clustered by the listed company. ^***^, ^**^ and ^*^ denote significance at the 1, 5, and 10% level, respectively. Firm controls include Size, SOE, ROA, Age, Leverage, TobinQ, Board, Indirector, Supervisor. Individual controls include Duality, CEO_age, CEO_edu.

Further dealing with the sample selection problem, the results demonstrate that CEO turnover has a statistically significant and positive impact on innovation performance, supporting the conclusion from baseline regression.

### Heterogeneity effects

This paper further discusses the heterogeneity of the effect of CEO turnover on corporate innovation at the CEO level and the firm level.

#### Heterogeneity of CEO characteristics

##### CEO turnover frequency

The explanatory variable in the baseline model is constructed based on the first-time CEO turnover of a listed company during the sample period. It is assumed that the innovation performance is only affected by this change event. However, some listed companies in the sample have changed their CEOs several times. A question naturally arises: Does a company with more times of CEO turnover have better innovation performance? According to the number of CEO turnovers, we further define the variable, *Multiple*, whether a listed company has multiple CEO turnovers, which equals one if the times of CEO turnover of a company are greater than 1, and equals zero otherwise. We then construct the interaction terms by multiplying the regressor of interest by the above variable, to perform the tests, and the estimation results are reported in columns (1) and (2) in Table 4. The estimated coefficients of interaction term indicate that there is no statistically significant difference between multiple changes and one change on subsequent innovation. In other words, the positive effect of listed companies with multiple CEO turnovers on innovation performance is no better than that of listed companies with one change. That is, CEO turnover can promote corporate innovation, while the effect does not depend on the frequency of the event. We guess that frequent CEO turnovers might cause operation unstable, which is not conducive to the continuous development of innovation activities.

**Table 4:** Heterogeneity effects of CEO characteristics.

	(1)	(2)	(3)	(4)	(5)	(6)
Dependent variables	Invention	Citation	Invention	Citation	Invention	Citation
Turnover	0.096^***^	0.089^***^	0.052	0.051	−0.013	0.211^***^
	(0.034)	(0.028)	(0.042)	(0.033)	(0.062)	(0.059)
Turnover × Multiple	−0.049	−0.014				
	(0.050)	(0.038)				
Turnover × Duality			0.116^*^	0.101^*^		
			(0.064)	(0.055)		
Duality			−0.145^*^	−0.118^*^		
			(0.079)	(0.065)		
Turnover × CEO_edu					0.136^*^	−0.154^**^
					(0.069)	(0.064)
CEO_edu					−0.124	0.091
					(0.107)	(0.083)
Firm control variables	Yes	Yes	Yes	Yes	Yes	Yes
Individual control variables	Yes	Yes	Yes	Yes	Yes	Yes
Firm fixed effect	Yes	Yes	Yes	Yes	Yes	Yes
Time fixed effect	Yes	Yes	Yes	Yes	Yes	Yes
Individual fixed effect	Yes	Yes	Yes	Yes	Yes	Yes
R-squared	0.724	0.458	0.724	0.459	0.724	0.459
Observations	15,151	15,151	15,151	15,151	15,151	15,151

A constant term is included but not reported. Robust standard errors in parentheses are clustered by the listed company. ^***^, ^**^ and ^*^ denote significance at the 1, 5, and 10% level, respectively. Firm controls include Size, SOE, ROA, Age, Leverage, TobinQ, Board, Indirector, Supervisor. Individual controls include Duality, CEO_age, CEO_edu.

##### CEO duality

Holding two positions (i.e., CEO and chairman) will make the power of listed companies more concentrated, thus affecting the innovation strategy. To investigate the heterogeneous effect of CEO duality, we add the interaction term of variable, *Duality*, and independent variable, *Turnover*, to the regression model. As shown in columns (3) and (4) in Table 4, the estimated coefficients of interaction term are positive and statistically significant, which means that the quantity and quality of innovation for listed companies with CEO duality will be significantly improved after the change event. However, the coefficient of variable, *Duality*, is statistically significant and negative, indicating that dual employment itself is not conducive to innovation. Overall, the aggregate effect of CEO duality on innovation performance is negative. Compared to companies without turnovers, CEO duality enhances the innovation for companies with CEO turnovers. We speculate that after the CEO turnover, the power concentration brought by the dual role will make innovation strategy easier to implement.

##### CEO education

Some studies find that the CEO’s education will affect the enterprise’s innovation activities (Islam and Zein, 2020). We discuss the heterogeneous effect brought by the change of CEOs with different degrees. We add the interaction term of the variable, *CEO_edu*, and the independent variable, *Turnover*, to the regression model. Columns (5) and (6) in Table 4 show that there is no statistically significant correlation between CEO education and innovation performance. The estimated coefficients of interaction term show that better CEO education will significantly improve the quantity of innovation, while reducing the quality of innovation. The possible reason is that the newly appointed CEO with better education focuses on R&D activities but ignores the innovation quality in the short-term innovation process.

#### Heterogeneity of company characteristics

##### Financing constraints

The output of innovation activities is highly uncertain and with risks, which makes innovation face financing constraints. Following Hadlock and Pierce (2010), this paper calculates the SA index to measure listed companies’ financing constraints. Add the variable and its interaction term with the explanatory variable to the econometric model. The results reported in columns (1) and (2) in Table 5 show no significant correlation between financing constraints and innovation quantity, but a statistically significant and negative correlation with innovation quality. For the samples with CEO turnover, high financing constraints significantly increase the quantity and quality of innovation. This finding may be because the new CEO focuses on innovation efficiency and can optimize innovation strategies when facing financing constraints.

**Table 5:** Heterogeneity effects of company characteristics.

	(1)	(2)	(3)	(4)	(5)	(6)
Dependent variables	Invention	Citation	Invention	Citation	Invention	Citation
Turnover	0.053	0.061^**^	−0.060	0.105^**^	0.068^*^	0.096^***^
	(0.037)	(0.031)	(0.051)	(0.042)	(0.037)	(0.031)
Turnover × FC	0.157^**^	0.105^*^				
	(0.069)	(0.054)				
FC	−0.114	−0.104^*^				
	(0.074)	(0.056)				
Turnover × High_tech			0.240^***^	−0.026		
			(0.063)	(0.052)		
High_tech			−0.037	0.034		
			(0.109)	(0.082)		
Turnover × Large_size					0.097	−0.027
					(0.065)	(0.048)
Large_size					−0.085	0.033
					(0.069)	(0.049)
Firm control variables	Yes	Yes	Yes	Yes	Yes	Yes
Individual control variables	Yes	Yes	Yes	Yes	Yes	Yes
Firm fixed effect	Yes	Yes	Yes	Yes	Yes	Yes
Time fixed effect	Yes	Yes	Yes	Yes	Yes	Yes
Individual fixed effect	Yes	Yes	Yes	Yes	Yes	Yes
R-squared	0.724	0.459	0.725	0.458	0.724	0.458
Observations	15,151	15,151	15,151	15,151	15,151	15,151

A constant term is included but not reported. Robust standard errors in parentheses are clustered by the listed company. ^***^, ^**^ and ^*^ denote significance at the 1, 5, and 10% level, respectively. Firm controls include Size, SOE, ROA, Age, Leverage, TobinQ, Board, Indirector, Supervisor. Individual controls include Duality, CEO_age, CEO_edu.

##### High-tech industry

The impact of CEO turnover on enterprise innovation can be different in various industries due to their strategic objectives. We refer to the industry classification of listed companies, divide the samples into high-tech and other industries, and discuss heterogeneity. As shown in columns (3) and (4) in Table 5, the positive impact of CEO turnover on the number of innovations is more prominent for companies in high-tech industries, while there is no significant difference in innovation quality. Short-sighted may be responsible for this fact where some enterprises in the high-tech industry merely emphasize the quantity of innovation rather than the quality value after CEO changes.

##### Enterprise size

Enterprise scale affects enterprise R&D resources, human capital, and is highly associated with innovation. We regard the median of the size of all observations as the benchmark, and define whether an observation is a large-size enterprise. As reported in columns (5) and (6) in Table 5, the coefficients of interaction term of CEO turnover and size of listed companies are not significant. The possible explanation for this result is that enterprises of different sizes will adjust their innovation strategies according to their conditions.

## Mechanisms

After establishing that CEO turnover can significantly promote both the quantity and quality of corporate innovation. This section attempts to reveal the mechanisms to understand the effect. This paper holds that CEO turnover may affect innovation through two channels: Reorganize the senior management team, and increase R&D investment.

### Top management team restructuring

To test the research hypothesis about organizational structure change, we follow Crutchley et al. (2002), to calculate the reorganization degree of the senior management team. The CEO turnover event itself also belongs to the reorganization of the senior management team, but this paper measures the reorganization of senior management team in a broader sense, including all directors, senior managers and supervisors. The calculation is shown in the following equation:


(3) $$RI_{t,t+1}=1-\left[\begin{array}{l} \frac{M_{t}-\#\left(S_{t}/S_{t+1}\right)}{M_{t}}\times \frac{M_{t+1}}{M_{t}+M_{t+1}}+\\ \frac{M_{t+1}-\#\left(S_{t+1}/S_{t}\right)}{M_{t+1}}\times \frac{M_{t}}{M_{t}+M_{t+1}} \end{array}\right]$$

where *RI*_*t*,*t*+*1*_ represents the reorganization value of the senior management team. The higher the value (close to 1) means a higher possibility of reorganization of the senior management team. *M_t_* represents the number of senior management teams in year *t*, and *M*_t+1_ represents the number of the senior management team in year *t +* 1. $\#\left(S_{t}/S_{t+1}\right)$ represents the number of members who belong to the senior management team in year *t* but not in year *t +* 1. $\#\left(S_{t+1}/S_{t}\right)$ represents the number of members who belong to the senior management team in year *t +* 1 but do not belong to the senior management team in year *t*. The calculation in the bracket measures the stability of the top management team.

This paper calculates the reorganization value of the senior management team of each company using the window of 1 year and 2 years, respectively. We use the calculated variables as the dependent variables, and regression results are reported in Table 6. We find that CEO turnover has a statistically significant and positive impact on the possibility of the senior management team restructuring, which confirms the previous speculation. In other words, CEO turnover is more likely to make the senior management team restructure, and then adjust the business and innovation strategy, to enable the enterprise to promote innovation performance. Therefore, the above estimation results confirm Hypothesis 2 that CEO turnover increases innovation by reorganizing senior management team.

**Table 6:** Top management team restructuring.

	(1)	(2)
Dependent variables	RI_t1	RI_t2
Turnover	0.049^*^	0.068^*^
	(0.026)	(0.041)
Firm control variables	Yes	Yes
Individual control variables	Yes	Yes
Firm fixed effect	Yes	Yes
Time fixed effect	Yes	Yes
Individual fixed effect	Yes	Yes
R-squared	0.245	0.423
Observations	10,223	8,387

A constant term is included but not reported. Robust standard errors in parentheses are clustered by the listed company. ^***^, ^**^ and ^*^ denote significance at the 1, 5, and 10% level, respectively. Firm controls include Size, SOE, ROA, Age, Leverage, TobinQ, Board, Indirector, Supervisor. Individual controls include Duality, CEO_age, CEO_edu.

### R&D investment

To verify the theoretical prediction about R&D adjustment, this paper uses two dependent variables, R&D expenditure and R&D personnel. The regression results in Table 7 show that the R&D expenditure and personnel are significantly higher for the treatment group after the change compared with the listed companies without CEO turnover. In other words, CEO turnover significantly promotes the investment of R&D expenses and R&D personnel, and then promotes the innovation output of the enterprise, which supports Hypothesis 3.

**Table 7:** R&D input.

	(1)	(2)
Dependent variables	R&D expenditure	R&D personnel
Turnover	0.002^***^	0.082^**^
	(0.001)	(0.041)
Firm control variables	Yes	Yes
Individual control variables	Yes	Yes
Firm fixed effect	Yes	Yes
Time fixed effect	Yes	Yes
Individual fixed effect	Yes	Yes
R-squared	0.294	0.474
Observations	8,577	2,557

A constant term is included but not reported. Robust standard errors in parentheses are clustered by the listed company. ^***^, ^**^ and ^*^ denote significance at the 1, 5, and 10% level, respectively. Firm controls include Size, SOE, ROA, Age, Leverage, TobinQ, Board, Indirector, Supervisor. Individual controls include Duality, CEO_age, CEO_edu.

## Conclusion

As the backbone of the enterprise, the CEO determines the behavior and performance of the enterprise to a certain extent. CEO turnover will change the strategic behavior of enterprises, and then have an impact on enterprise innovation. Taking all listed companies in China as the research object, this paper uses the staggered DID model to investigate the impact of CEO turnover on corporate innovation. First, we find that CEO turnover has positive effects on both the innovation quantity and innovation quality. Specifically, compared with the listed companies without CEO turnover, the quantity of innovations and the quality of innovation of the listed companies with CEO turnover increases by 9.5 and 8.9% after the turnover event. Second, the heterogeneity test results show that in terms of the quantity of innovations, CEO turnover has a more prominent role in promoting the listed companies in duality, better educated CEOs, high financing constraints, and in high-tech industries. As for innovation quality, CEO turnover has a greater effect on enterprises in the high-tech industry and has a weaker impact on enterprises with better educated CEO. Third, the mechanism tests show that CEO turnover promotes innovation by driving the senior management team reorganization and increasing R&D expenditure and personnel investment.

The limitations of this study mainly reside in only investigating the empirical evidence of the impact of CEO turnover on innovation, even though the study conducts a bunch of tests to ensure the robustness of findings. Moreover, although the data used in this study is one of the most representative firm-level data in China, the sample of listed companies is not able to reflect all other firms like small and medium-sized enterprises. To tackle the above two issues, future research is suggested to construct a theoretical model to analyze this problem, which is hopeful to undercover the mechanisms theoretically. Besides, further research is encouraged to explore this topic using other firm-level data to understand this relationship comprehensively.

## Data availability statement

The raw data supporting the conclusions of this article will be made available by the authors, without undue reservation.

## Author contributions

SS: developed the study concept and drafted the manuscript. HJ: performed testing and analysis, provided critical revisions, and participated in the final revision of the manuscript. SS and HJ: contributed equally to this work. All authors contributed to the article and approved the submitted version.

## Acknowledgments

We are grateful for financial support from China’s National Social Science Funding (Grant number: 20CXW014).

## Conflict of interest

The authors declare that the research was conducted in the absence of any commercial or financial relationships that could be construed as a potential conflict of interest.

## Publisher’s note

All claims expressed in this article are solely those of the authors and do not necessarily represent those of their affiliated organizations, or those of the publisher, the editors and the reviewers. Any product that may be evaluated in this article, or claim that may be made by its manufacturer, is not guaranteed or endorsed by the publisher.
