# Supplementary material for: CEO turnover and corporate innovation: What can we learn from Chinese listed companies
Source: Front Psychol. 2022 Jul 20;13:874907. doi: 10.3389/fpsyg.2022.874907 (PMC9346083; doi:10.3389/fpsyg.2022.874907)
Supplement: Supplementary file 1 [file Data_Sheet_1.docx]

### Appendix

## Correlations

Table A1 Pairwise correlations

|  | Size | SOE | ROA | Age | Leverage | TobinQ | Board | Indirector | Supervisor | Duality |
| --- | --- | --- | --- | --- | --- | --- | --- | --- | --- | --- |
| Size | 1 |  |  |  |  |  |  |  |  |  |
| SOE | 0.299^***^ | 1 |  |  |  |  |  |  |  |  |
| ROA | -0.030^***^ | -0.137^***^ | 1 |  |  |  |  |  |  |  |
| Age | 0.186^***^ | 0.058^***^ | -0.101^***^ | 1 |  |  |  |  |  |  |
| Leverage | 0.475^***^ | 0.321^***^ | -0.410^***^ | 0.158^***^ | 1 |  |  |  |  |  |
| TobinQ | -0.344^***^ | -0.268^***^ | 0.314^***^ | 0.014^*^ | -0.399^***^ | 1 |  |  |  |  |
| Board | 0.263^***^ | 0.300^***^ | -0.009 | -0.040^***^ | 0.194^***^ | -0.198^***^ | 1 |  |  |  |
| Indirector | 0.080^***^ | -0.076^***^ | 0.001 | 0.066^***^ | -0.018^**^ | 0.066^***^ | -0.369^***^ | 1 |  |  |
| Supervisor | -0.148_***_ | -0.276^***^ | 0.068^***^ | -0.028^***^ | -0.183^***^ | 0.159^***^ | -0.191^***^ | 0.119^***^ | 1 |  |
| Duality | 0.240^***^ | 0.394^***^ | -0.065^***^ | -0.002 | 0.205^***^ | -0.181^***^ | 0.362^***^ | -0.130^***^ | -0.167^***^ | 1 |

## Robustness tests

### Additional firm-level controls

Venture capital can act as a phased financing tool to support enterprises to carry out R&D and then affect innovation performance (e.g., Lerner and Nanda, 2020). We further control whether listed companies obtain venture capital (*VC*). Columns (1) and (2) in Table A2 show no statistically significant correlation between venture capital and innovation. After controlling venture capital, CEO turnover still has a statistically significant and positive impact on the quantity and quality of innovation.

Some studies find that enterprises with different capital intensities have different innovation behaviors (e.g., Autor et al., 2020). In order to control this factor, we calculate the capital-labor ratio (*Capital_labor*) of each observation. The results in columns (3) and (4) in Table A2 show no significant correlation between capital intensity and innovation performance. After controlling the capital-labor ratio, CEO turnover positively impacts the innovation quantity and quality at the 1% level.

Intangible assets reflect an enterprise’s knowledge pool to a certain extent and are thus closely related to enterprise innovation (e.g., Graham et al., 2018). We calculate the proportion of intangible assets to total assets (*Intangible*), and control it in the regressions to reduce this potential effect. The estimated results in columns (5) and (6) in Table A2 show that intangible assets are positively correlated with the quantity of innovation, while there is no statistically significant correlation with the quality of innovation. In addition, CEO turnover has a statistically significant and positive impact on innovation performance, consistent with the benchmark regression results.

### Industry- and regional-level fixed effects

During enterprises’ innovation activities, some industry regulations and local policies might exert an impact. Industrial policies include the degree of industry competition and industrial parks (e.g., Aghion et al., 2005; Lu et al., 2019). Regional characteristics and policies contain economic development, foreign investment attraction, innovation subsidy policy and intellectual property protection (e.g., Jiang et al., 2022; Ang et al., 2014). Since these factors are difficult to measure and control one by one, we try to control the two-way fixed effects to reduce the potential biases, and the estimations are reported in Table A3. Based on the baseline regression, columns (1) and (2) control the industry-time fixed effects, columns (3) and (4) control the province-time fixed effects, and columns (5) and (6) control the two pairs of fixed effects. After controlling the potential unobservable variables at the industry- and regional- levels, the estimated results are in line with baseline regression results.

Table A2 Additional firm-level controls

|  | (1) | (2) | (3) | (4) | (5) | (6) |
| --- | --- | --- | --- | --- | --- | --- |
| Dependent variables | Invention | Citation | Invention | Citation | Invention | Citation |
| Turnover | 0.090^**^ | 0.087^***^ | 0.094^***^ | 0.085^***^ | 0.087^**^ | 0.088^***^ |
|  | (0.039) | (0.032) | (0.034) | (0.028) | (0.035) | (0.029) |
| VC | -0.016 | -0.004 |  |  |  |  |
|  | (0.054) | (0.046) |  |  |  |  |
| Capital_labor |  |  | 0.002 | 0.001 |  |  |
|  |  |  | (0.001) | (0.001) |  |  |
| Intangible |  |  |  |  | 0.036^**^ | 0.006 |
|  |  |  |  |  | (0.014) | (0.009) |
| Firm controls | Yes | Yes | Yes | Yes | Yes | Yes |
| Individual controls | Yes | Yes | Yes | Yes | Yes | Yes |
| Firm fixed effect | Yes | Yes | Yes | Yes | Yes | Yes |
| Time fixed effect | Yes | Yes | Yes | Yes | Yes | Yes |
| Individual fixed effect | Yes | Yes | Yes | Yes | Yes | Yes |
| R-squared | 0.724 | 0.458 | 0.725 | 0.459 | 0.723 | 0.452 |
| Observations | 15,151 | 15,151 | 15,114 | 15,114 | 14,748 | 14,748 |

Note: A constant term is included but not reported. Robust standard errors in parentheses are clustered by the listed company. ^***^, ^**^ and ^*^ denote significance at the 1%, 5%, and 10% level, respectively. Firm controls include Size、SOE、ROA、Age、Leverage、TobinQ、Board、Indirector、Supervisor. Individual controls include Duality、CEO_age、CEO_edu.

Table A3 Industry- and province-level fixed effects

|  | (1) | (2) | (3) | (4) | (5) | (6) |
| --- | --- | --- | --- | --- | --- | --- |
| Dependent variables | Invention | Citation | Invention | Citation | Invention | Citation |
| Turnover | 0.074^**^ | 0.087^***^ | 0.095^***^ | 0.088^***^ | 0.075^**^ | 0.087^***^ |
|  | (0.034) | (0.028) | (0.037) | (0.029) | (0.036) | (0.029) |
| Firm controls | Yes | Yes | Yes | Yes | Yes | Yes |
| Individual controls | Yes | Yes | Yes | Yes | Yes | Yes |
| Firm fixed effect | Yes | Yes | Yes | Yes | Yes | Yes |
| Individual fixed effect | Yes | Yes | Yes | Yes | Yes | Yes |
| Industry-time fixed effect | Yes | Yes | No | No | Yes | Yes |
| Province-time fixed effect | No | No | Yes | Yes | Yes | Yes |
| R-squared | 0.729 | 0.473 | 0.725 | 0.461 | 0.731 | 0.475 |
| Observations | 15,133 | 15,133 | 14,205 | 14,205 | 14,175 | 14,175 |

Note: A constant term is included but not reported. Robust standard errors in parentheses are clustered by listed company. ^***^, ^**^ and ^*^ denote significance at the 1%, 5%, and 10% level, respectively. Firm controls include Size、SOE、ROA、Age、Leverage、TobinQ、Board、Indirector、Supervisor. Individual controls include Duality、CEO_age、CEO_edu.

## Figure and Table for PSM

Figure A1 PSM covariate comparison

Table A4 PSM output variable comparison results

|  | (1) | (2) | (3) | (4) |
| --- | --- | --- | --- | --- |
| Variables | Treat group | Control group | Difference | t-statistic |
| Panel A. Original sample（before PSM） |  |  |  |  |
| Invention | 1.248 | 0.717 | 0.532 | 21.17 |
| Citation | 0.720 | 0.479 | 0.241 | 16.90 |
| Panel B. Matched sample（after PSM） |  |  |  |  |
| Invention | 1.249 | 1.057 | 0.191 | 6.18 |
| Citation | 0.720 | 0.616 | 0.104 | 5.35 |
